# Self-inhibition of growth and allelopathy through volatile organic compounds in *Fusarium solani* and *Aspergillus fumigatus*

**DOI:** 10.1371/journal.pone.0308383

**Published:** 2024-08-27

**Authors:** Takae Takeuchi, Takahito Suzuki, Tomoko Kimura, Masato Kiuchi

**Affiliations:** 1 Division of Materials and Manufacturing Science, Osaka University, Suita, Japan; 2 Department of Chemistry, Nara Women’s University, Nara, Japan; 3 Cerast Laboratory Co. Ltd, Setagaya, Japan; Leibniz-Institut fur Naturstoff-Forschung und Infektionsbiologie eV Hans-Knoll-Institut, GERMANY

## Abstract

Microbial volatile organic compounds (VOCs) emitted from fungi are known as their secondary metabolites from environmental sources. However, their physiological roles remain to be unclear. Even though the roles are still unknown, VOCs are deliberately released to convey information to both homologous and non-homologous organisms. We investigated the effects of single VOCs (hexanal, benzaldehyde, heptanal, 2-ethyl-1-hexanol, 3-octanone, 2-undecanone, 3-octanol, 2-Phenylethanol, 2-phenyl-2-propanol, phenylbenzaldehyde, 2-pentadecanone, β-trans-bergamotene, β-bisabolene, 2-methyl-5 -(1-methylethyl)pyrazine) on the fungal growth. In parallel, application of the co-culturing system in a growth chamber allowed free gas and VOCs exchange between emitter colonies of *Fusarium solani* and *Aspergillus fumigatus*, or between colonies of different growth stages of the same species. Distinct self-inhibition occurred by the emitters of fungal growing colonies against receiver ones on the stage of conidial germination or against the younger colonies at an earlier stage in both fungi. Similarly, the phenomenon of allelopathy appeared to work between growing colonies of *F*. *solani* and the germinating conidia or young colonies of *A*. *fumigatus* or vice versa. Solid phase microextraction—gas chromatography/mass spectrometry revealed VOCs compounds of each fungi. In *F*. *solani*, hexanal and benzaldehyde appeared to be significant inhibitors for colony growth. Benzaldehyde inhibited filamentous growth but not conidial germination. In *A*. *fumigatus*, heptanal seemed to be an equivalent effector. The inhibitory effect of benzaldehyde was more distinct on the *A*. *fumigatus* conidial germination than its filamentous growth.

## Introduction

The abilities of fungal spores to germinate and develop highly polarized hyphae enable filamentous fungi to colonize on new substrates rapidly and efficiently. An intricate network of these cells forms the mycelial colony. The processes to colony formation respond to both various types of environmental cues and endogenous regulation. Since many cases to the colony formation occur on solid substrates, signals from the atmosphere seem to be efficient natural stimuli of the formation [1]. The first step of colony formation is the conidial germination, in which favorable growth conditions permit the progress into hyphal filament formation. Once their spores (or asexual conidia) are made, they will be dispersed throughout the air but do not germinate before they reach a suitable substrate. Many fungal spores exhibit a crowding effect [2] in which the spores contain a prepackaged self-inhibitor that prevents germination under crowded, high-cell-density conditions. Self-inhibitors of germination have been reported in several species, including *F*. *oxysporum* [3], and can be located outside the spore cells. These compounds also can be removed by washing with water [4, 5].

Nonanoic acid was presented as one of the sporostatic factors inhibiting spore germination in *F*. *oxysporum* [6]. In liquid culture of *F*. *oxysporum*, inhibition of germination worked at the spore density of 10^6^ per mL or more. Another type of self-inhibitors may be fungal volatile organic compounds. By using static headspace analyses, Chitarra et al., [7] reported 1-octen-3-ol as the compound appeared to block the germination process at different developmental stages of the conidia (swelling and germ tube formation) in *Penicillium paneum*. Microorganisms, including fungi and bacteria, emit volatile organic compounds (VOCs) as their metabolites [8]. The emission of VOCs seems to be an effective candidate for an indicator of the occurrence of fungal growth in the environment [9–11]. Several literatures have regarded more than 200 organic compounds as VOCs [11–13]. These compounds consist of metabolites originated solely from microbial metabolism [13] and also include, in a broad sense, catabolites from environmental sources such as chloroanisoles [14]. Some VOCs are known to be generally synthesized in most of the fungi and the others as strain- or species-specific [15]. Recently, VOCs have been suggested to affect human health, causing lethargy, headache, and irritation to the eyes and mucous membranes of the nose and throat [16]. A new method has been developed to collect analytes in the headspace of the sample, allowing for the analysis of low concentrations of VOCs using gas chromatography combined with mass spectrometry (GC/MS) [9, 17]. Solid phase micro extraction (SPME) is a passive sampling method for aqueous and gaseous matrices that uses different types of fiber coatings for analyte extraction and concentration. Nilsson et al., [18] evaluated SPME’s usefulness for headspace analysis of VOCs emitted by five *Penicillium* species.

Therefore, these compounds are used more and more frequently for the search for hidden mold growth [19–21]. Examinations of profiles of VOCs may also be applicable to a notification of fungal occurrence in the environment [22]. When we were investigating fungi damaging the wall paintings of ancient mound tombs in Nara, Japan, we discovered that fungi including *A*. *fumigatus and F*. *solani* co-habited in the ancient mound tombs. We collected VOCs of several fugal species, including *A*. *fumigatus and F*. *solani* by SPME and analyzed by GC/MS [23]. In this work, we found some of the VOCs of these fungi possessing growth inhibitory effect on the surrounding colonies of different species as well as of the same one. The physiological and ecological roles of VOCs will be discussed. We consider the term allelopathy, in this article on fungal behaviour, to be ’the influence of VOCs emitted by a fungus on the behaviour of other fungi, e.g. the effect on other fungi of promoting or inhibiting mycelial growth or spore formation’.

## Materials and methods

### Strains, media, and growth conditions

*F*. *solani* NBRC31093 was purchased from Department of Biotechnology, National Institute of Technology and Evaluation, Kisarazu, Chiba Prefecture, Jpn. *A*. *fumigatus* IFM40822 was kindly given to us by Dr. Koji Yokoyama at Medical Mycology Center, Chiba University, 1-8-1 Inohana, Chuo-ku, Chiba, 260–8673, Jpn. A chemically defined dextrose-salt Czapek-Dox (CZ) medium [24] was used as the basal growth medium with slight modifications as the following constituents; 0.5 g sodium citrate, 3 g NaNO_3_, 1g K_2_PO_4_, 0.5 g MgSO_4_・7H_2_O, 0.5 g KCl, 0.01 g Fe(II)SO_4_・7H_2_O, 30 g dextrose and 1 liter of distilled water. For VOCs analysis, 2-mL volume of the CZ agar medium in the bottom of a 20 mL-screw vials (75.5 x 22.5 mm) and caps with PTFE/Sil septa (for COMBI PAL GC analysis, CTC Analytics) was used for cultivation of fungal conidia. The potato dextrose agar medium (PDA, Difco) was also used as a growth medium for collecting conidia. For observation of the effect of the test compound on fungal growth and conidia germination, CZ agar media were allowed to solidify in the bottom lids of sterile 35- by 15-mm petri dishes. Suitable number of conidia was a center inoculated onto the agar media. Both the petri dish base carrying inoculated media and a designated quantity of the test compound, which was dispensed by a sterile 18-gauge needle into the bottom of a 0.5-mL microcentrifuge tube with a small round opening petri plate were located at 15-mm apart from each other, in the 150- by 25-mm petri dish that was then covered with a lid. This large petri dish was finally sealed with Parafilm. This system allowed free gas inside the large one. Cultures were incubated at 28°C and in 99.8% relative humidity in the dark for the appropriate time periods (see Results), and the diameter of each colony on a small petri plate was measured. Observations with the VHX-500 digital microscope with 16 bits high dynamic range (Keyence Corp. Osaka, Jpn) allowed us to examine a series of events, consisting of conidial germination, colony growth and the subsequent formation of daughter conidia. For examining interactions between the two organisms via VOCs, the application of the co-culturing system allowed free gas or volatile exchange between colonies inside the large petri dish without lids while preventing direct colony contact, as described below. Culture plates were incubated at 28°C and in 99.8% relative humidity in the dark for the appropriate time periods. In some experiments, culture plates were sealed with a thin membrane filter (MILLI WRAP, Millipore Co. Bedford MA) during incubation, in order to ensure not to be contaminated from conidia released from each of the petri plates. Colony growth was estimated by measuring diameters of colonies on the solid medium using a vernier caliper, according to Brancato and Golding [25].

### Co-culturing systems allowing free gas or volatile exchange between fungal colonies while preventing direct colony contact

The co-culturing system [26] was applied to allow free gas or volatile exchange between colonies while preventing direct colony contact in a closed chamber. The 3X and 6X treatments were constructed with the large petri dish (150- by 25-mm) as the chamber containing either three or six small petri plates (35- by 15-mm) without lids carrying the 3-days culture of the emitter organism. Another small petri dish without a lid was inoculated with conidia (1 x 10^2^ to 1 x 10^5^ per plate). Cultures in the chamber were incubated for five days at 28°C and the colony diameter of the receiver strain was measured during incubation.

### Volatile organic compounds analysis

Conidia (2 x 10^3^) were spotted on the 2-mL CZ solid medium in a 20 mL-screw vial (GL Sciences Inc. Rolling Hills Estates, CA, USA) and the mouth of the vial was covered with aluminum foil. In this case, the air is not blocked and oxygen supply is possible. Then, the vial was incubated for the particular incubation period between 1 and 9 days at 28°C as according to Takeuchi et al. [23].

Fungi grow in an environment where oxygen is present. After a certain period of incubation, the VOCs generated are replaced by fresh air. Incubate for 24 hours with the lid of the 20 mL-screw vial closed and with no air exchange. This traps the VOCs generated in 24 hours. We have also observed that during 24 hours of incubation, the vials are not oxygen-depleted in the 20 mL-screw vial. The 20 mL-screw vial is heated at 80°C for 15 min to stop fungal activity. The fungus may have died. However, it does not release any more VOCs. Under these conditions, the amount of VOCs released in 24 hours was determined. A solid-phase microextraction (SPME) fiber coated with divinylbenzene, polydimethylsiloxane and carboxen (SPELCO Ltd., Cat. No.57298-U) is used for collection of emitted VOCs. The purpose of using SPME to adsorb VOCs is to determine the composition ratio of gas molecules in the vial. Adsorption for 15 minutes, 2 hours and 24 hours was tested. The amount of substance adsorbed at each time is greater over time. However, the composition ratios of the adsorbates obtained were the same for all adsorption conditions, within the margin of error. Therefore, it was found that the composition ratios obtained after 15 minutes of adsorption can be used as representative values of the experimental results.

The emitted VOCs during the next one day incubation period were collected using SPME. According to this measurement, we could take the rate of emission of an individual VOC per day. The other method was to measure accumulated amounts of VOCs in the headspace in the vial during the incubation period. The mouth of each vial was sealed with a screw cap at the start of cultivation. The SPME was inserted into the vial tube and VOCs were adsorbed for 15 min at 80°C. The SPME was then removed from the vial tube, and desorbed in the injection port (200°C) of the gas chromatograph-mass spectrometer system (GCMS-QP2010, Shimadzu Co., Kyoto, Jpn). Detection of VOCs was performed using a quadruple mass spectrometer equipped with an electron ionization-ion source. The temperature of EI ion source is 200°C. Measurements were performed on three replicates. Appropriate controls for medium, plastic ware, and accidental contaminants were conducted.

In order to measure the concentrations of each VOC in the screw vials, we performed parallel measurements on the other screw one including each 1 μl methanol solution of the serially diluted VOC substance from commercial sources. A linear calibration curve on each of the VOC enabled us to convert arbitrary units of ion peak density to parts-per notation, such as ppm and ppb. Calibration was performed on heptanal, hexanal and benzaldehyde.

The pH was measured using a Horiba Ion Sensitive Field Effect Transistor Electrode, which is capable of measuring the surface of agar media. At least three measurements were taken in each condition. At least three vials per time were also used for the measurements. Calibration curves were drawn using the least-squares method, in accordance with normal statistical methods.

## Results

### Characterization of colony growth in a closed chamber in both *F*. *solani* and *A*. *fumigatus*

In this study, our first interest was whether *F*. *solani* evolved efficient mechanisms to utilize self-synthesizing VOCs, for regulation of their growth on the solid medium. In order to examine the occurrence of regulation in colony growth via gas phase, we applied the co-culturing system consisting of small culture-plates (1 or 6 ones, each with 35 mm in diameter, without lids) in one large petri plate (150 mm in diameter) as the growth-chamber. This system intended to cut off the gas exchange between the inner phase of the chamber and its external environment. *F*. *solani* visible colonies appeared at approximately 1-day intervals after the start of cultivating the 10^4^, 10^3^ and 10^2^ conidial inoculations in this order on each of the six culture-plates in the chamber. The colonies continued their growth linearly for more three days in those cultures after their appearance. The rate of the increase in diameter of the concentric growing area was 11.0 ± 0.7 mm day^-1^. Colony growth increased in size and finally its peripheral region extended outside of the edge of the plates. No significant growth inhibition appeared to occur during 8, 9 or 10-days on each of the cultures starting 10^4^, 10^3^ or 10^2^ conidial inoculations. The pH of each plate started at 6.5 and reached 7.7 after 10 days-incubation. The single culture plate in the chamber gave final pH value as 8.1. This difference between the pH values may be due to that of produced amounts of some metabolites. Experiments were conducted with one and six small culture plates contained in one large petri dish. In each, the oxygen available for culture was six times different. In addition, the concentration of dissipated CO_2_ is 1/6. However, the growth rates of both are the same.

Therefore, neither oxygen content nor carbon dioxide one in the headspace of the chamber appeared to cause significant effect on the *F*. *solani* colony growth in this situation. On the other hand, in the case of *A*. *fumigatus* colony growth, the simultaneous incubation of six small culture plates in the chamber revealed visible colonies at approximately 0.5-days intervals in the 10^6^, 10^5^, 10^4^ and 10^3^ conidial inoculations in this order, and the colony growth continued linearly (6.2 ± 0.5 mm in diameter day^-1^) for 4 days after the start of cultivation. However, the further incubation ceased colony growth by the 5-days period in the 10^3^ inoculations, by the 6-days one in the 10^4^ ones, by the 7-days one in the 10^5^ ones, and by the 8-days one in the 10^6^ ones after the start of cultivation. At the 8-days period, the surface area of the colony of 10^5^, 10^4^ or 10^3^ ones decreased to 79 ±8, 60 ±6 and 46 ±5% in this order, compared to that of the 10^6^ one. The maximum amount of colony growth appeared to decrease with the number of inoculated conidia from 10^6^ to 10^3^. In both 10^3^ and 10^4^ ones at the 8-days period, color of the central zone of individual colonies turned reddish-brown, differently from deep-green of those of 10^5^ and 10^6^ ones. Turning color on the surfaces of these *A*. *fumigatus* colonies appeared to indicate the immature conidia production, after the observations with the digital microscope. The pH of each one changed from 6.5 at the start of cultivation to 4.5 after 8 to 10 days-incubation. The growth rate of *A*. *fumigatus* in a single plate in the chamber showed approximately the same with that of the six ones during the early 7 days-incubation period and gave final pH value as 5.0 after 10 days-incubation. The maximum amount of colony growth reached the level similar to the six culture plates of the 10^6^ inoculations, irrespective of the number of inoculated conidia. Turning color phenomenon did not occur in the single culture plate. Either or both decreased content of the oxygen or increased one of the carbon dioxide appeared to work as the limiting factor for the colony growth of *A*. *fumigatus* after the 7 days-incubation period.

VOCs emissions decrease after day 9, possibly due to quorum sensing (QS) conditions.

### The regulation via gas-phase occurred on both conidia germination and early colony growth in *F*. *solani* as well as in *A*. *fumigatus*

**Fig 1:**
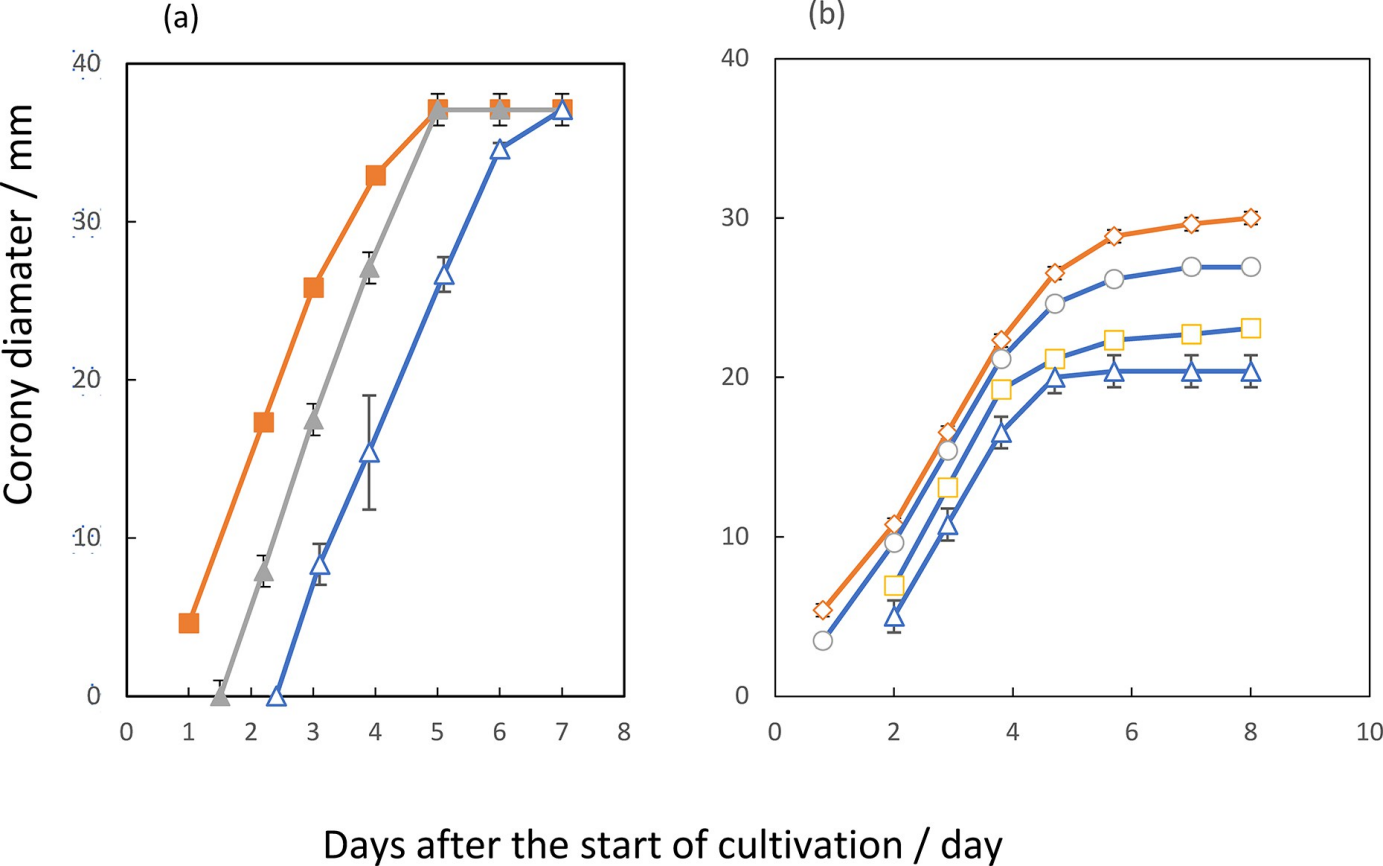
Colony growth curves of *F*. *solani* NBRC31093 and *A*. *fumigatus* IFM40822 in the closed chamber constructed with the large petri dish (150- by 25-mm). In *F*. *solani*, each of six petri plates without lids (35- by 15-mm) was inoculated with 1 x 10^3^ conidia (Δ), 1 x 10^4^ ones (□), or 1 x 10^5^ ones (○) and incubated at 28°C. The vertical line shows the diameter of the colony. The data presented are mean±SD from at least three independent experiments.

The process of fungal colony growth included two stages, the conidial germination and the following filamentous hyphal formation. The colony showed a concentric circular growth pattern rather than irregular one. Some self-regulatory mechanisms seemed to work during the colony growth. One of the rate-limiting factors for initiating the growth appeared to be the numbers of conidia inoculated at the start of cultivation (Fig 1).

**Fig 2:**
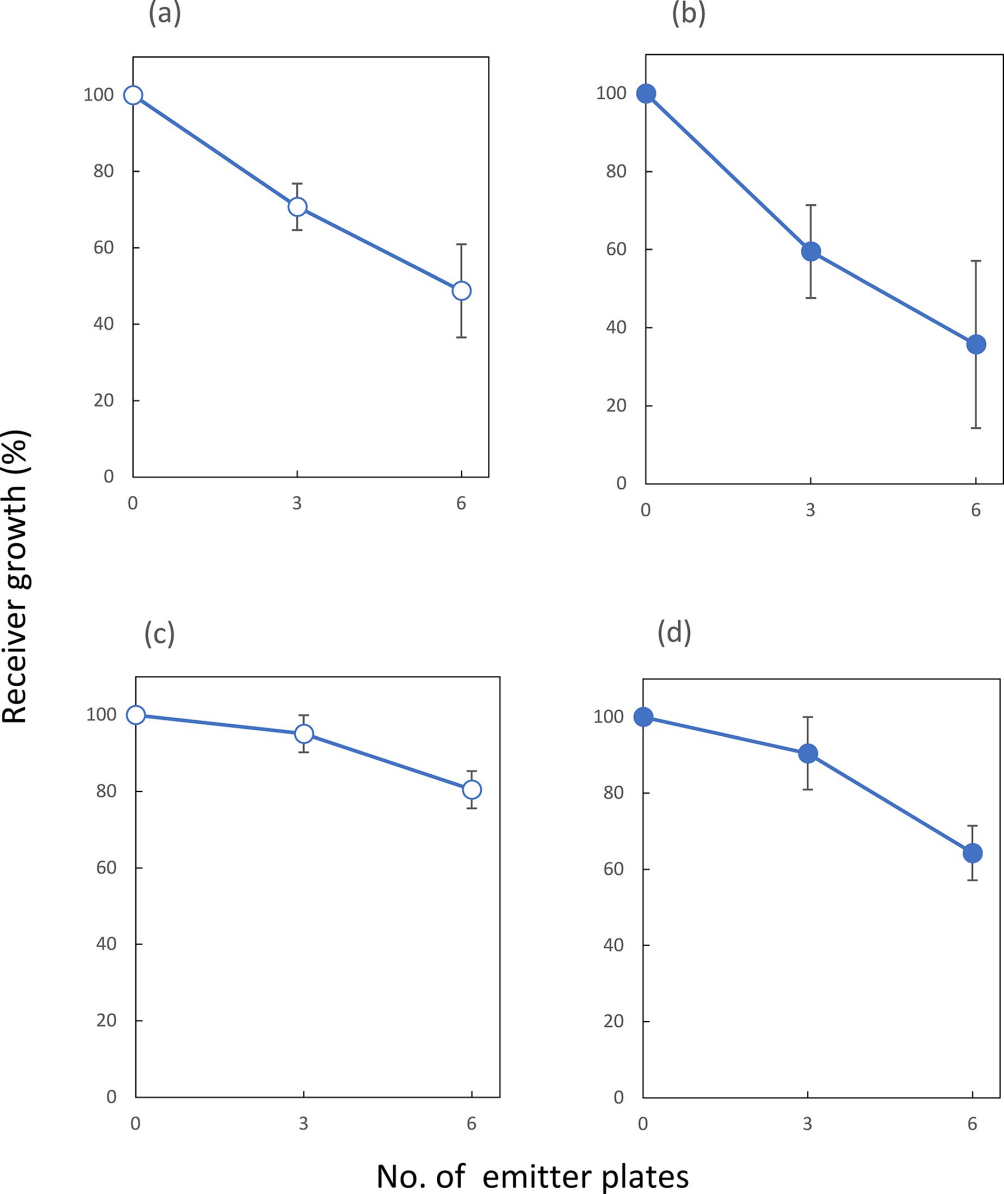
Inhibition of mycelial growth depending on the dose of headspace gases. (a) A receiver plate was inoculated with 2 x 10^3^ *A*. *fumigatus* IFM40822 conidia and the surrounding emitter plates previously cultured for 2-days after the inoculation with 2 x 10^3^ ones, were co-cultured in a closed chamber for seven days at 28°C. The receiver growth represents % colony growth area, compared to that without emitter plates. The horizontal axis shows the number of surrounding emitter plates. All of the data presented are mean ±SD from at least three independent experiments. (b) A receiver plate was inoculated with 1 x 10^3^ *F*. *solani* NBRC31093 conidia and the surrounding emitter plates previously cultured for 2-days after the inoculation with 1 x 10^3^ ones, were co-cultured in a closed chamber for seven days at 28°C. The receiver growth represents in the same way with (a). (c) One *F*. *solani* receiver plate, inoculated with 2 x 10^3^ conidia, was co-cultivated for seven days at 28°C in the sealed chamber, with 0, three or six emitter plates of *A*. *fumigatus*, which had been incubated for 2-days after the inoculation of 2 x 10^3^ conidia. The receiver growth represents in the same way with (a). (d) One *A*. *fumigatus* receiver plate, inoculated with 1 x 10^3^ conidia, was co-cultivated for seven days at 28°C in the sealed chamber, with 0, three or six emitter plates of *F*. *solani*, which had been incubated for 2-days after the inoculation of 2 x 10^3^ conidia. The receiver growth represents in the same way with (a).

We tried to find another one affecting the growth by applying the growth experiments in a closed vessel, in terms of VOCs entities in the headspace. To investigate this problem, we applied co-culturing experiment consisting of two types of culture plates, one inoculated conidia and the others in the stage of filamentous hyphal growth, set in the same growth chamber. Since we assumed these latter plates emitting some VOCs, into the headspace of the chamber, and named the plates as emitter ones. On the other hand, the former plate seemed to be influenced by the VOCs and was named as a receiver one. In the case of *F*. *solani*, self-inhibition appeared to efficiently and reproducibly occur when the receiver was set at the center of the chamber and inoculated 1 to 2 x 10^3^ conidia, and combined with six emitter ones of 3-days cultures, which originally inoculated with 2 x 10^3^ ones and having the colony-diameter of approximately 10 mm (Fig 2A). In this condition, each of emitter ones showed colony growth increasing in size and finally extending its peripheral region outside of the edge of the plate by the 5-days period. Besides, the inhibition scarcely took place if the number of inoculated conidia of the receiver, exceeding 10^4^ or those of the number of each emitter plate over 10^4^ ones. Thus, via the headspace VOCs emitted from *F*. *solani* colonies under the filamentous growth phase, the self-inhibition appeared to work on the receiver restrictedly in both stages of conidial germination at a low density and of the following early colony formation. Namely, the fungus indicated the most sensitive stage against self-inhibitors to be the early stage of colony growth.

The degree of inhibition against the receiver appeared to decrease if subtracting the number of surrounding emitter plates from six to three (Fig 2A). In this figure, per cent of growth represented the upper limit of growth as the final diameters of the receiver colonies after 8 days-incubations, compared to those of control cultures consisting of seven inoculated plates cultivated simultaneously in the chamber. In this experiment, the colony growth rates and the maximum level of colony diameters of the three emitters were almost the same with those of the six ones. Based on these findings, *F*. *solani* seemed to emit some growth-inhibitors in a growth-stage dependent manner.

In the other filamentous fungus, *A*. *fumigatus*, we examined activities of VOCs emitted from the growing colony by inoculating conidia in the receiver plate with conidia numbers from 10^3^ to 10^5^ and surrounding three or six emitter plates in various growth stages. Growth inhibition reproducibly occurred when the one receiver plate contained 1 to 3 x 10^3^ conidia and the six emitter ones of 2-days cultures originally inoculated with 1 x 10^3^ conidia. In the case of three emitter ones, the receiver obtained 40% growth, and aerial hypha formation appeared to cease, and that of six ones, the receiver showed 30% growth and the basal filament elongation into the agar layer to stop, compared to those of control cultures consisting of seven inoculated plates cultivated simultaneously in the chamber (Fig 2B). The growth inhibition did not work when the numbers of inoculated conidia exceeded 10^4^ on the receiver plate. However, maximum levels of colony growth of *A*. *fumigatus* emitter plates were 40% less than that of *F*. *solani* on the 6-day cultivation period.

This fact also supported the notion that an oxygen deficiency was another limiting factor leading to self-inhibition of the colony growth in this organism. Therefore, via the headspace gas emitted from *A*. *fumigatus* colonies under linear colony-growth phase, the self-inhibition by VOCs appeared to work on the receiver within the early stages of colony growth, the condition of which was the low density of inoculated conidia such as 10^3^ and the proceeding filamentous growth during the periods at 2 to 3 days after the start of cultivation. The other growth-limiting factor working at the later stage of colony growth at more than 4 days cultivation may be the oxygen deficiency in this organism.

### Mutual inhibition or allelopathy between *F*. *solani* and *A*. *fumigatus*

The emission of VOCs by a fungal organism provoked another question whether volatiles-mediated interactions can result in selective advantage to some community members in microbial ecosystems. The *F*. *solani* emitter, grown on CZ media, paired with an inoculated plate of the other receiver *A*. *fumigatus* and vice versa under the co-culturing experiments. Headspace VOCs, released from the *F*. *solani* emitter plates, appeared to give inhibitory effects on the colony growth of the *A*. *fumigatus*, parallel with increased numbers of the emitter ones (Fig 2C). The combination of the number of *A*. *fumigatus* conidia, about 1 x 10^3^, inoculated on a receiver plate and *F*. *solani* emitters, cultivated for 2 days after inoculating approximately 2 x 10^3^ conidia on each of the plates, gave reproducible results. The growth rate of an *A*. *fumigatus* colony alone in the chamber gave 6.0 ± 0.5 mm in diameter day^-1^ during the period from 2- to 4-days incubation. However, additional *F*. *solani* six emitter plates of the 2-days cultures brought about 60% inhibition (2.2 ± 0.4 mm in diameter day^-1^) of the growth rate of the *A*. *fumigatus* reeiver during the 2- to 4-days incubation period. The case of three *F*. *solani* platesas emitters gave 37% inhibition in the rate (3.8 ± 0.4 mm in diameter day^-1^ from these experiments, diameter day^-1^).

On the other hand, the combination of *A*. *fumigatus* emitter and a single plate of *F*. *solani* receiver, significant inhibition also occurred on the receiver, as far as various numbers of inoculated conidia of each of the two organisms were examined. Among these experiments, reproducible and clear inhibitions occurred in the combination of the 2 days-emitter plates after inoculating approximately 3 x 10^3^ conidia and a receiver one with 1 to 2 x10^3^ conidia (Fig 2D). In this case, the colonies of the *A*. *fumigatus* emitters ceased their growth by 8-days period. The growth inhibition of the *F*. *solani* receiver did not occur when the number of inoculated conidia of the receiver exceeded 10^4^, or that of the emitter was more than 10^4^ or less than 5 x 10^2^. From these experiments, one could presume that the common growth inhibitors may work on both self-inhibition and mutual one.

### Volatile analysis of headspace gases of *A*. *fumigatus* and *F*. *solani* cultures

**Fig 3:**
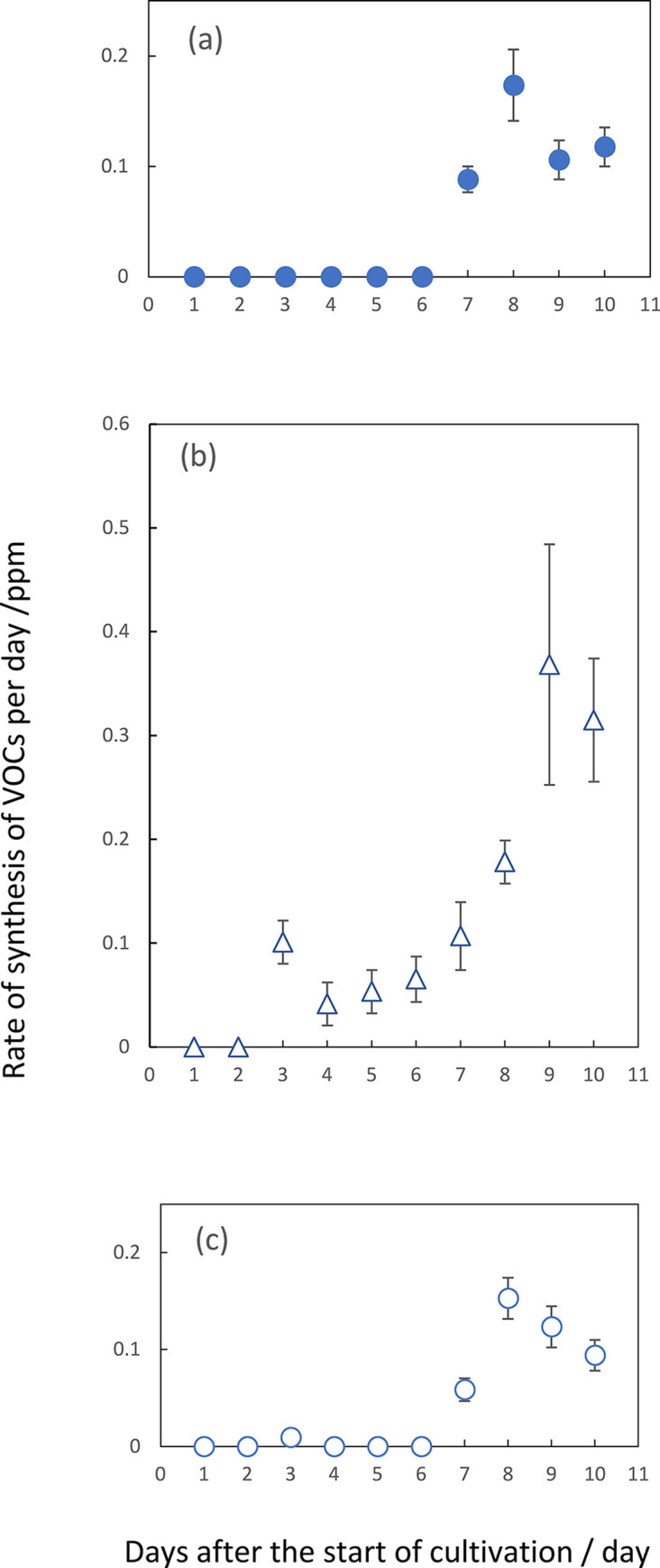
The synthesis rate of VOCs in the head space of vials of *F*. *solani* during 10 days-incubation periods. Each volatile compound was measured by SPME/GC/MS. From the intensity of total-ion current chromatograms, the concentration of each volatile was expressed as ppm. A linear calibration curve on each of the volatile enable us to convert arbitrary units of ion peak density to parts-per million (ppm). Symbols; hexanal (a, ●), benzaldehyde (b, Δ) and 2-pentadecanone (c, ○). The data presented are mean ±SD from at least three independent experiments.

Headspace gases obtained from each two mL-solid culture of the *F*. *solani* NBRC31093 strain were subjected to GC/MS analyses. After changing the headspace with fresh air, the accumulated volatiles during a one-day incubation period, analyzed on the next day, gave us the synthesis rate of VOC per one day. *F*. *Solani* NBRC31093 grown on CZ media emitted, at least, ten compounds; 2-ethyl-1-hexanol, hexanal, 3-octanone, 2-undecanone, 3-octanol, 2-phenylethanol, 2-phenyl -2-propanol, benzaldehyde, phenylbezaldehyde and 2-pentadecanone. Among them, hexanal and 2-pentadecanone appeared to increase their emission at the concentrations of 0.1 to 0.2 ppm (Fig 3A and 3B). The maximum rate of benzaldehyde emission reached 0.4 to 0.5 ppm (Fig 3C). The other ones, 2-ethyl-1-hexanol, 2-phenylethanol and 2-phenyl -2-propanol, 3-octanone, 2-undecanone and 3-octanol gave their concentrations at several ten ppb. The emitted amount of 2-phenylethanol was several ppb. Likewise, *A*. *fumigatus* IFM40822 grown on CZ media emitted, at least, seven ones; 2-ethyl-1-hexanol, 3-octanone, 2-undecanone, heptanal, β-trans-bergamotene, β-bisabolene and 2-methyl-5- (1-methylethyl) pyrazine. Among them, heptanal and β-trans-bergamotene showed its emission rate at 0.1 to 0.2 ppm (Fig 3A and 3B). Heptanal appeared to increase its amount gradually. A characteristic feature was the short time emission of β-trans-bergamotene, which showed a peak at the 8 day after the start of cultivation (Fig 3B), the period of which approximated to the start of conidia formation. The maximum emission of the other substances were several ten ppb.

**Fig 4:**
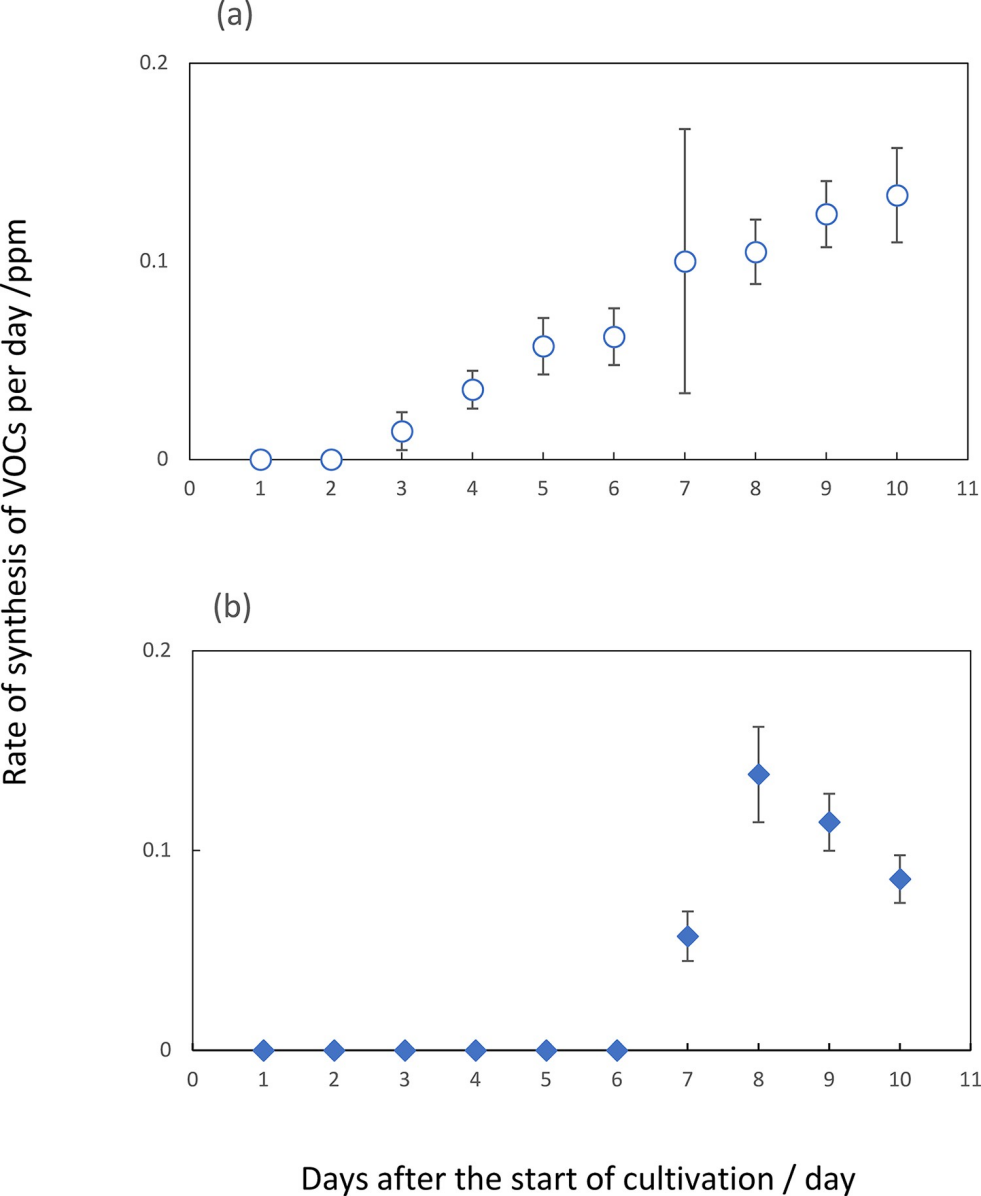
The synthesis rate of VOCs in the head space of vials of *A*. *fumigatus* during 10 days-incubation periods. The concentration of each volatile was expressed as ppm as described in the Fig 3. Symbols; heptanal (a, ○) and β-trans-bergamotene (b, ◆). The data presented are mean ±SD from at least three independent experiments.

**Fig 5:**
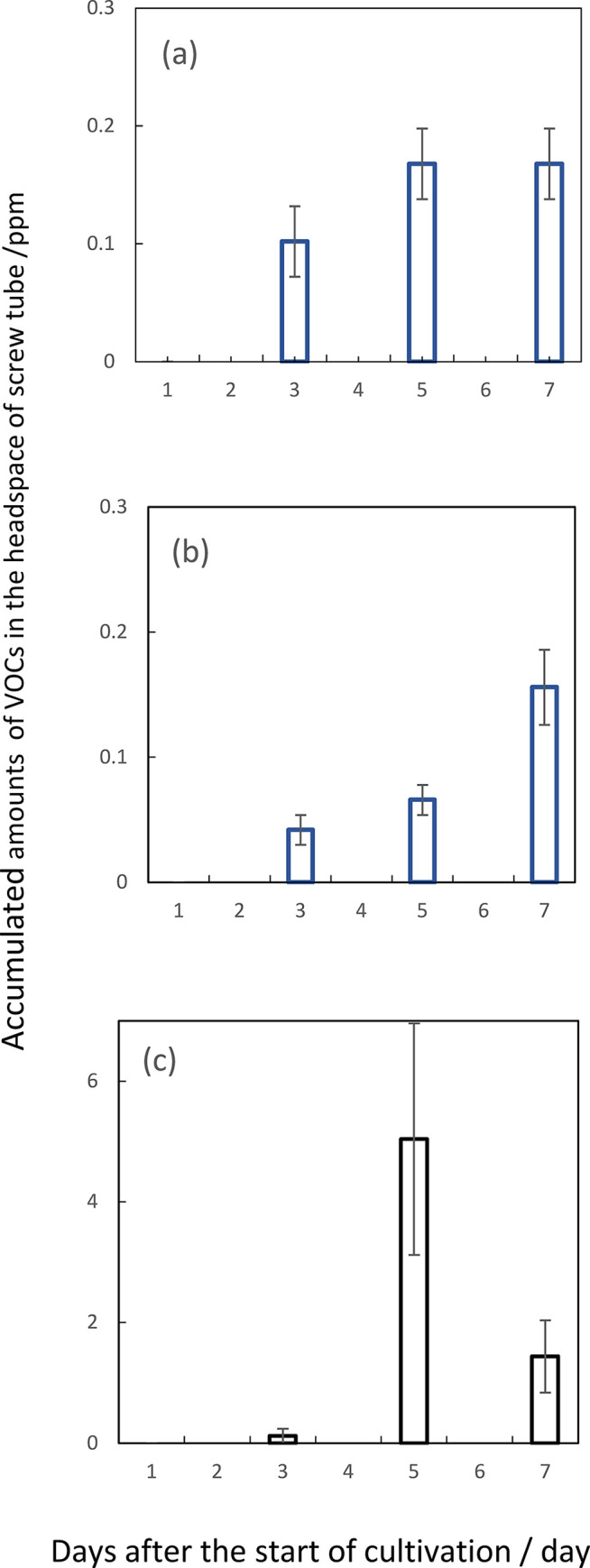
Accumulated amounts of major VOCs in the head space of vials of *F*. *solani* and *A*. *fumigatus* at 3-, 5- and 7-days after the start of cultivations. The concentration of each volatile was expressed as ppm as described in the Fig 3. Hexanal (a) and benzaldehyde (b) in *F*. *solani* cultures or heptanal (c) in *A*. *fumigatus* ones. The data presented are mean ±SD from at least three independent experiments.

The other way to measure an accumulated amount of VOCs in *F*. *solani* revealed the net quantity of synthesized hexanal in the headspace of the screw tube after the start of cultivation (Fig 5A). On the other hand, the accumulated amount of benzaldehyde in this organism appeared to increase with the lapse of incubation time (Fig 5B). Compared to the profile of these accumulated amounts of hexanal, the data of its synthesis rate were behind time (Fig 4B). Similarly in *A*. *fumigatus*, the net amount of heptanal showed its maximum at fifth day after the start of cultivation (Fig 5C). This period was also at least two or three days earlier than that showing the maximum rate of emission of this volatile.

In the two organisms, an efficient self-inhibition or mutual one occurred by the emitter one of three to five-days cultures. Therefore, hexanal in *F*. *solani* and heptanal in *A*. *fumigatus* seemed to be a volatile candidate for growth regulators. Furthermore, benzaldehyde, emitted by *F*. *solani*, seemed to be another candidate for inhibiting the colony growth of both organisms. With regards to 2-pentadecanone, its net amount increased with the lapse of incubation time. Since no apparent inhibition with the exogenous volatile one occurred in the colony growth of both organisms, as described below, the profile data were omitted.

### Examination of volatile candidates for activity of self-inhibitor and/or allelopathy

In order to narrow down the candidates for the self-inhibitor of colony growth, we examined physiological activities of the seven VOCs (hexanal, 2-ethyl-1-hexanal, 3-octanol, benzaldehyde, 3-octanone, phenylacetaldehyde and 2-phenyl-2-propanol) on *A*. *fumigatus* and the ten (2-ethyl-1-hexanal, benzaldehyde, 3-octanol, 3-octanone, phenylacetaldehyde, 2-phenyl-2-propanol, hexanal, heptanal, 2-phenylethanol and 2-undecanone) on *F*. *solani*. For estimations of the activity of each VOC, we inoculated conidia of the *A*. *fumigatus* or *F*. *solani* on three sets of CZ agar plates (35- by 15-mm) in a 150- by 25-mm polystyrene petri dish as a chamber and intended to distinguish inhibitory effects of candidates between conidial germination and hyphal elongation. First time, we examined hexanal, heptanal, benzaldehyde and 2-pentadecanone, since the concentrations of these substances reached at the level of ppm in the headspace of screw vials.

**Fig 6:**
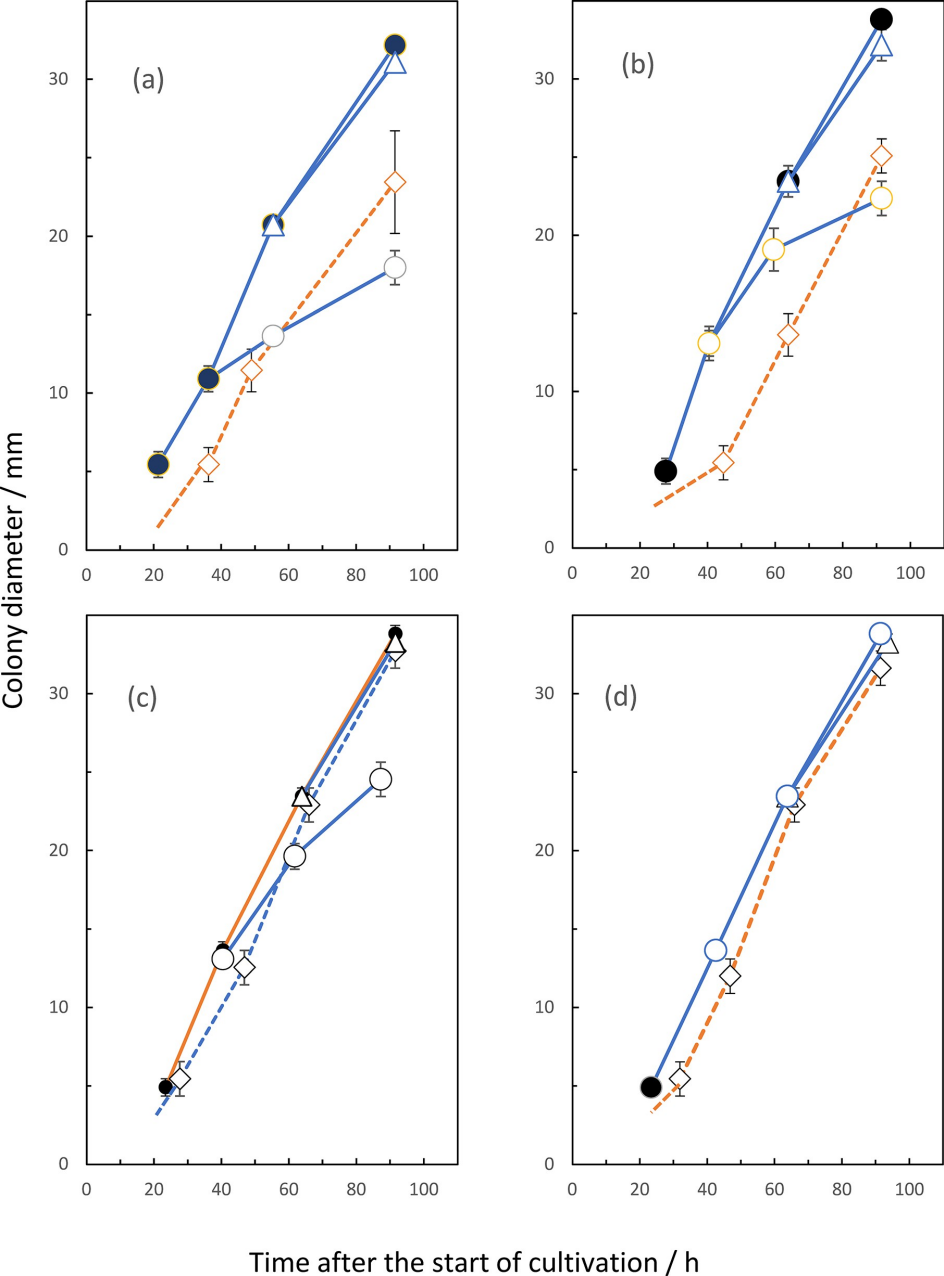
Inhibition of *F*. *solani* growth by exogenous VOCs. Colony growth was estimated by measuring diameters of six colonies, each on the solid medium in a petri plates without lids (35- by 15-mm), inoculated with 2 x 10^3^ conidia at the start of cultivation. These six culture plates were set in a closed petri dish (150- by 25-mm) as the chamber. The chamber was applied to allow 0.4 mg amount of commercially available VOC, so as to the initial concentration of each VOC as 1 ppm. Exogenous VOCs were hexanal (a), heptanal (b), benzaldehyde (c), and 2-pentadecanone (d). Symbols; control cultures without addition of VOC (●); the addition of each VOC at 0 h (◊), approximately at 40 h (○) or near at 60 h (Δ).

**Fig 7:**
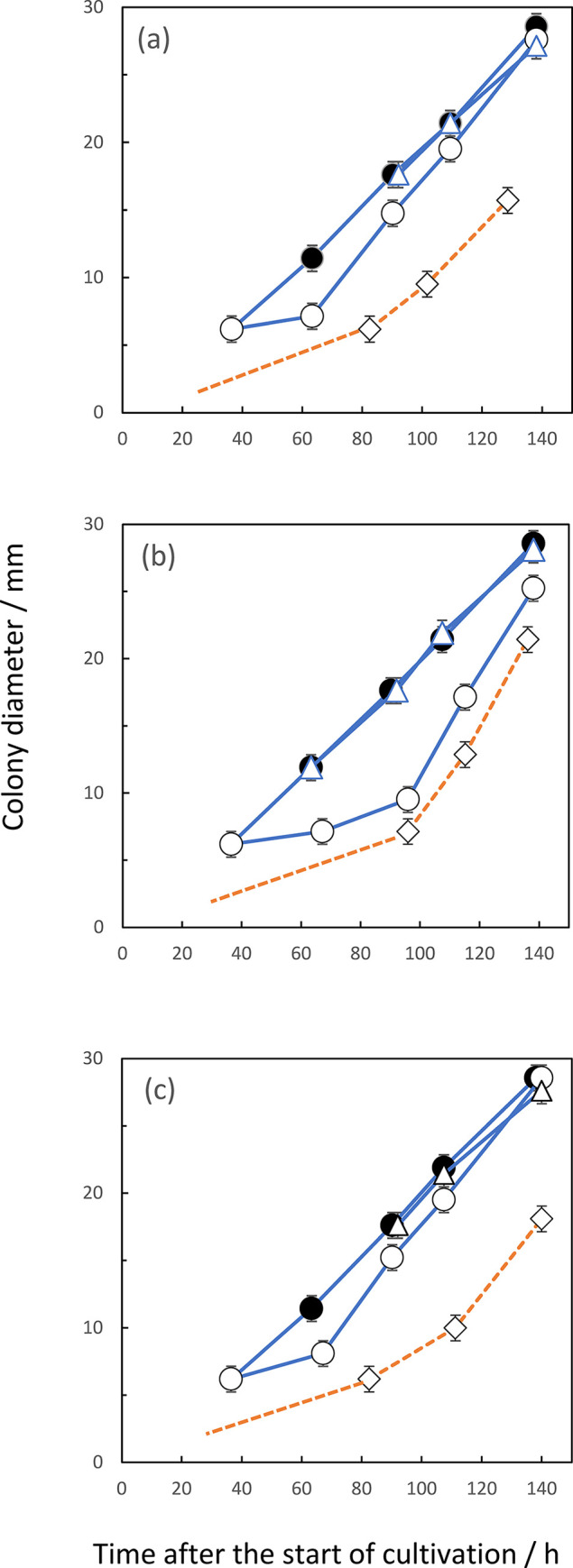
Inhibition of *A*. *fumigatus* growth by exogenous VOCs. Monitoring colony growth and culturing methods were the same with those in Fig 6. Exogenous VOCs were hexanal (a), heptanal (b), and benzaldehyde (c). Symbols; control cultures without addition of VOC (●); the addition of each VOC at 0 h (◊), at 36 h (○) or near at 64 h (Δ).

For this purpose, we estimated the initial concentrations of each volatile in the headspace (approximately 4.4 x 10^3^ mm^3^ volume) as 1 ppm under the assumptions of both that the compounds would diffuse completely and that no significant absorption would occur at the surface of plastic dishes. However the absorption would occur at the surface of plastic dishes and by the medium, the amount of absorption is assumed to be sufficiently low because the absorption is only at the top surface of materials. The chamber was applied to allow 0.4 mg amount of commercially available VOC at various incubation periods from 0 to 96 h in the *F*. *solani* receiver or 0 to 140 h in the *A*. *fumigatus* one, after the start of cultivation. If an exogenous VOC, supplemented at 0 h, brought about some inhibition at the germination stage, visible colonies appeared after a significant lag period, compared to the non-supplemented control. Similarly, if the addition of the VOC was performed at some period of colony growth, inhibition of filamentous growth occurred. Figs 6 and 7 represented the occurrence of such inhibitions.

When the culture plates of *F*. *solani* were applied for the receiver (Fig 3), hexanal and heptanal caused inhibitions of both germination and colony growth of an early stage. Benzaldehyde also brought about the similar inhibition of colony growth. However, the germination process appeared not to be affected. Neither 2-pentadecanone nor β-trans-bergamotene seemed to affect any inhibition at any stage. From the date on the rate of synthesis of VOCs, the maximum rate of hexanal at 6 to 7 days after the start of cultivation and benzaldehyde at 9 to 10 days. However, the data on the accumulated amounts of these two VOCs revealed the maximum ones of hexanal at 5 days and that of benzaldehyde at around 7 days after the start of cultivation. This apparent discrepancy between the rate and accumulation data will be discussed later. From the latter accumulation data, the main self-inhibitor of *F*. *solani* could be attributed to be hexanal. Benzaldehyde also seemed to work as a self-inhibitor of colony growth but not of germination in this organism. Fig 6A also suggested the occurrence of the gradual increase in the tolerance to the inhibition of two VOCs in accordance with the progress of colony growth. These two substances also seemed to work as the growth inhibitors against *A*. *fumigatus*. Likewise, some mismatching existed between the periods of maximum emission rate and that of accumulated amounts of heptanal in *A*. *fumigatus*. According to the latter accumulation data indicating the maximum amount at around 5 days after the start of cultivation (Fig 5C), heptanal strongly suggested as the main self-inhibitor of colony growth in *A*. *fumigatus*. This substance also worked as the growth inhibitor against *F*. *solani* during co-culturing in the same chamber. Besides, exogenous benzaldehyde inhibited both germination and filamentous growth of *A*. *fumigatus*. This fact suggested benzaldehyde as the allelochemical substance derived from *F*. *solani*. The other exogenous VOCs, including 2-ethyl-1-hexanol, 2-phenylethanol and 2-phenyl -2-propanol, 3-octanone, 2-undecanone and 3-octanol in F. solani and 2-ethyl-1-hexanol, 3-octanone, 2-undecanone, β-bisabolene and 2-methyl-5- (1-methylethyl) pyrazine in *A*. *fumigatus*, did not show any significant inhibition on the colony growth of both organisms as far as their concentrations were prepared as 0.1 ppm. Since actual concentrations of these substances in the headspace of the culture chamber were less than dozen ppb, these did not seem to contribute as the main growth inhibitors against the two fungal species.

## Discussion

Three VOCs (hexanal, heptanal, and benzaldehyde) found during the self-inhibitory experiment demonstrated one common characteristic. It was the aldehyde, and especially the former two substances may be produced during decomposition of fatty acid hydroperoxides following a peroxidation attack. Benzaldehyde may be the conversion product of phenylalanine initiated by an aminotransferase. Significant differences existed on our data on the maximum values between the rates of synthesis and the accumulated amounts of these aldehydes in the headspace of the culture tubes. The former rates were the amount of *de novo* synthesis per day, obtained during one day-incubation after changing the headspace gas within the tube with fresh air. According to this measuring method, fungal colonies always exposed themselves enough amounts of oxygen at the first time during one-day incubation. This experimental condition almost seemed to be equivalent to the cultivation in the opened vessels. On the other hand, the condition giving the latter accumulated ones was the typical example in the closed-culture vessels with a gradual decrease in oxygen concentrations during the process of cultivation. Therefore, the active period of the emission of growth inhibitors by the emitter colonies in the closed chamber almost appeared to be equal during the period showing the maximum amount of the VOCs inhibitor accumulated in the headspace of the chamber. These findings also suggested the rates of synthesis of these aldehydes as the possible parameters of fungal cell density in the opened-culture vessels, and probably in the closed ones as well. Some correlations may exist between the concentrations of oxygen or presumable carbon dioxide and the rates of emission of these aldehydes. Further studies, including the controlling experiments of the concentrations of oxygen as well as carbon dioxide during fungal culturing will clarify these problems.

Song et al. [27] described on the inhibition of fungal activity caused by hexanal vapor, in which the 4.1-μmol·L^–1^ treatment inhibited growth of *Penicillium expansum* and *Botrytis cinerea* completely during the 48-h exposure period. The concentration of the hexanal in their treatment almost equaled with 0.41 ppm. In our experiment, exogenous hexanal at an initial concentration as 1 ppm in the plastic chamber significantly inhibited the growth of *F*. *solani* as well as *A*. *fumigatus*. Even if one considered the possible absorption of vapor hexanal by the chamber at some degree, the net concentrations of hexanal working as the growth inhibitor in our chamber experiments may be almost the same order with those of the hexanal vapor treatment. Another volatile component of many fruits, especially after wounding, (E)-2-hexenal, the lipoxygenase-lyase product, has been implicated in plant pathogen defense and observed to inhibit fungal spore germination and both inhibit and promote germ tube elongation in a concentration-dependent manner [27, 28]. In the case of *A*. *nidulans*, 2-buten-1-ol (or crotyl alcohol) was found exerting dose-dependent regulatory effects on conidial formation and colony growth of *A*. *parasiticus* [26]. We also checked the effect of 2-buten-1-ol on spore germination and colony growth in the two fungal strains in this study. The results showed inhibitory effects of this compound against the *A*. *fumigatus* colony growth. Therefore, the effective concentrations of exogenous VOCs for colony-growth inhibition, including hexanal, benzaldehyde and heptanal in the headspace of our culture chambers seemed to be fully reasonable.

Conidia normally germinate under favorable growth conditions, but also produce signals that inhibit conidial germination in overcrowded conditions, termed auto-inhibitors [29], which ensure efficient substrate colonization. Nonanoic acid was a sporostatic factor inhibiting spore germination in *F*. *oxysporum* [6]. The fungus *P*. *paneum* emitted an eight-carbon oxylipin (8CO) 1-octen-3-ol as a specific germination auto-inhibitor attributing the role of inducing micro cycle conidiation in spores, which had undergone germination under crowding conditions [7]. Recent studies on *Trichoderma* spp. showed that 1-octen-3-ol and other 8COs such as 3-octanone and 3-octanol were produced in conidiating cultures and interestingly, induced conidiation in non-conidiating neighboring cultures [30]. On the other hand, from our investigations on the conidial germination of both *F*. *solani* and *A*. *fumigatus*, we found starting periods of colony growth dependent on the number of inoculated conidia, namely the more numbers, the least durations of the periods, as far as the inoculating size was less than 10^5^. Rather there might be some substance working as an accelerator of conidia germination in both organisms. Since we could not detect any volatile compound as a candidate for accelerating germination at an early stage after the inoculation, some nonvolatile compounds might work as such the accelerator. We are now in progress to detect such candidate substances with liquid chromatography–mass spectrometry (LC-MS).

The fungal colonies of the two organisms in this study appeared to emit VOCs inhibitors during the late stage of their growth, equivalent to the period by which the filamentous growth proceeded at high cell density. In this respect, the three substances, hexanal, heptanal and bezaldehyde, seemed to behave as quorum sensing molecules [31], just like γ-heptalactone, which have been described in *A*. *nidulans* [32]. However, we should also consider the other possibility called the competition strategy, aiming the capture nutrients and niche occupation that had been described in microorganisms [33]. Another possibility of the role of self-inhibitory substances may be the signal for apical dominance, whereby the growing tip of fungal colonies is dominant and appears to suppress the formation of other tips or lateral branches in the immediate vicinity of the tips [34, 35].

Many of the VOCs reported in this study are either derivatives of intermediates of fungal primary metabolites or the secondary ones. These low-molecular-weight metabolites often have potent physiological activities, such as providing protection against other inhabitants in their ecological niche [36]. The secondary metabolism is commonly associated with sporulation processes in microorganisms including fungi [37]. Secondary metabolites associated with sporulation can be placed into three broad categories: metabolites that activate sporulation, for example, the so-called precocious sexual inducer, an endogenous mixture of hydroxylinoleic acid moieties produced by *A*. *nidulans* [38, 39] or pigments required for sporulation structures, for example, melanins required for the formation or integrity of both sexual and asexual spores and overwintering bodies [40]; and toxic metabolites secreted by growing colonies at the approximate time of sporulation, for example, the biosynthesis of some deleterious natural products, such as mycotoxins [41]. An interesting finding in this study was the short time emission of β-trans-bergamotene in *A*. *fumigatus*, which showed a significant peak at the period, equivalent to the starting one of conidial formation. As far as we examined, exogenous β-trans-bergamotene apparently did not bring any physiological change in mycelial growth. However, the volatile profiling seemed to provide the occurrence of intracellular chemical development linked to morphological one, as reported in *A*. *parasiticus* [42]. Thus, we will need further investigations to clarify the roles of this sesquiterpene compound.

Allelopathy was studied for major to minor fungi in co-culture. The impact of VOCs generated by major fungi on minor fungi was discussed. Recently, Chippendale et al. [43] and Neerincx et al. [44] have reported that the composition of the VOCs generated by one fungus to the other during co-cultivation can change. Further studies are needed as fungi show complex responses with regard to allelopathy.

Fungi and bacteria pose the severe threat to housing and furniture as well as cultural heritage materials [16, 45, 46]. Above all, fungi are predominant groups causing the devastating damages, because of their wide range of growth temperatures and humidity as well as their abilities to produce various degradation enzymes and metabolites [47]. Once their spores (or asexual conidia) are made, they will be dispersed throughout the air and attaching to new places, followed by promotion of germination into filamentous offspring by water condensation [46, 48]. The occurrence of visible fungal colonies makes it too difficult to suppress spreading of the organism due to the dispersal of uncountable numbers of offspring spores. In 2001, the bestiary of the Lascaux cave in France was contaminated with a white fungus, *Fusarium solani* [45, 49]. And in the same year, contamination of the fungus was also found on the mural paintings in the Takamatsu-zuka tumulus located at the village of Asuka, Nara Prefecture, Japan [50]. The fungus is the soil-borne pathogen for plants as well as for the man and animals.

Detection of fungal emergence at an earlier stage would allow for reducing risks of the contamination, and the fungal VOCs will be good environmental indices for this purpose.

## Supporting information

S1 Fig The synthesis rate of VOCs in the head space of vials of *A*. *fumigatus* during 10 days-incubation periods. Raw data of Fig 4 are presented.
(TIFF)

S1 Table Colony growth curves of *F*. *solani* NBRC31093 and *A*. *fumigatus* IFM40822 in the closed chamber constructed with the large petri dish (150- by 25-mm). Raw data of Fig 1 are presented.
(PDF)

S2 Table Inhibition of mycelial growth depending on the dose of headspace gases. Raw data of Fig 2 are presented.
(PDF)
