# Supplementary figures and images for: Self-inhibition of growth and allelopathy through volatile organic compounds in Fusarium solani and Aspergillus fumigatus
Source: PLoS One. 2024 Aug 27;19(8):e0308383. doi: 10.1371/journal.pone.0308383 (PMC11349182; doi:10.1371/journal.pone.0308383)

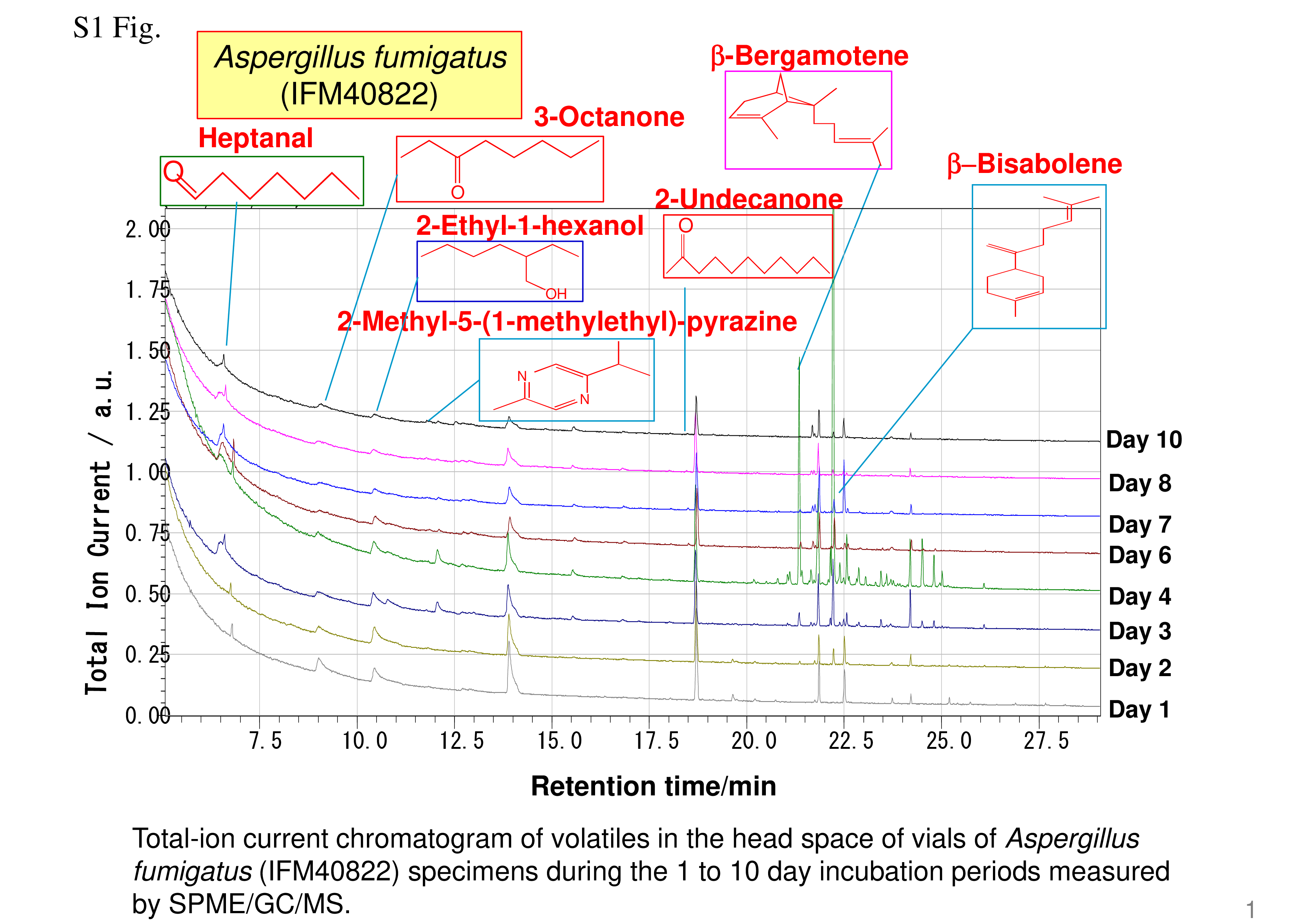

Supplement: S1 Fig — Raw data of Fig 4 are presented. (TIFF) [file pone.0308383.s001.tiff]
