# Supplementary material for: Self-inhibition of growth and allelopathy through volatile organic compounds in Fusarium solani and Aspergillus fumigatus
Source: PLoS One. 2024 Aug 27;19(8):e0308383. doi: 10.1371/journal.pone.0308383 (PMC11349182; doi:10.1371/journal.pone.0308383)
Supplement: S1 Table — Raw data of Fig 1 are presented. (PDF) [file pone.0308383.s002.pdf]

S1 Table.

(a)	Days after the start of cultivation / days	Corony diameter / mm					
○	1	6	5	4.5	4.5	5	4.5
	2.2	20	18	16	17	17	18
	3	28	22	23	25	25	24
	4	34	32	29	30	30	32
	5	34	32	28	29	29	29
	6	35	35	30	30	28	30
	7	35	35	30	30	28	30
□	1.5	0	0	0	0	0	0
	2.2	13	6	8	8	4	7
	3	21	14	17	16	14	16.5
	4	32	26	25	28	22	25
	5	35	35	35	35	35	35
	6	35	34	35	33	35	35
	7	35	35	35	35	35	35
△	2.2	0	0	0	0	0	0
	3	5.5	2	3.5	2	1	1
	4	16	11	17	13	9.5	12
	5	29	25	30	24	20	23
	6	35	34	35	35	33	35
	7	35	35	35	35	35	35
(b)							
▽	0.8	5	5	6	5.5	6	5.5
	2	11	11	11	10.5	11.5	10.5
	2.9	16	16	16.5	16	16.5	17
	3.8	22	23	23	24.5	21	23.5
	4.7	27	27	27.5	27	27	28
	5.7	28	30	30.5	30	29.5	31
	7	29	39	30.5	30	29.5	31
	8	31	34	31	35	32	33
○	0.8	3	4	3	2	4	4
	2	10.5	9.5	10.5	11	10.5	10
	2.9	16	16.5	16	15	15	16
	3.8	20	19.5	19	19.5	20	19.5
	4.7	25	24.5	25	25	24.5	25
	5.7	27	28.5	26	25	26.5	26
	7	29	30	28	27.5	27.5	28
	8	32	32	31	31.5	31	31
□	2	7	6.5	8	7.5	6.6	7.5
	2.9	13	13	13	14	14	13
	3.8	20	19.5	19	19.5	20	19.5
	4.7	22	21	21	22	21	21.5
	5.7	23	22.5	23	22.5	23	24
	7	23	22.5	24	23	23	20
	8	25	26	25	20	20	24
△	2	5	5	4.5	4.5	5	5
	2.9	11	12	10	9	10.5	11
	3.8	16	17	16	17	16	16
	4.7	20.5	21	20	20.5	21.5	21
	5.7	20.5	21.5	20	20	20.5	21
	7	20	22	20	21.5	21	22.5
	8	21	22	20	21	21	22.5
