# Supplementary material for: Self-inhibition of growth and allelopathy through volatile organic compounds in Fusarium solani and Aspergillus fumigatus
Source: PLoS One. 2024 Aug 27;19(8):e0308383. doi: 10.1371/journal.pone.0308383 (PMC11349182; doi:10.1371/journal.pone.0308383)
Supplement: S2 Table — Raw data of Fig 2 are presented. (PDF) [file pone.0308383.s003.pdf]

S2 Table.

	No. of emitter plates	Corony diameter / mm			Average of corony diameter / mm	Receiver growth(%)
(a)						
○	0	35	35	35	35.0	100.0
	3	24	26	27	25.7	73.3
	6	16	26	13	18.3	52.4
(b)						
●	0	35	35	35	35.0	100.0
	3	25	18	18	20.3	58.1
	6	9	15	16	13.3	38.1
(c)						
○	0	35	35	35	35.0	100.0
	3	32	35	34	33.7	96.2
	6	28	27	27	27.3	78.1
(d)						
●	0	35	35	35	35.0	100.0
	3	32	31	33	32.0	91.4
	6	25	21	25	23.7	67.6
